# Supplementary material for: Preparation and In Vitro Evaluation of Chitosan-g-Oligolactide Based Films and Macroporous Hydrogels for Tissue Engineering
Source: Polymers (Basel). 2023 Feb 11;15(4):907. doi: 10.3390/polym15040907 (PMC9962061; doi:10.3390/polym15040907)
Supplement: Supplementary file 1 [file polymers-15-00907-s001.zip › polymers-2194371-supplementary.pdf]

Supporting Information:

A B C

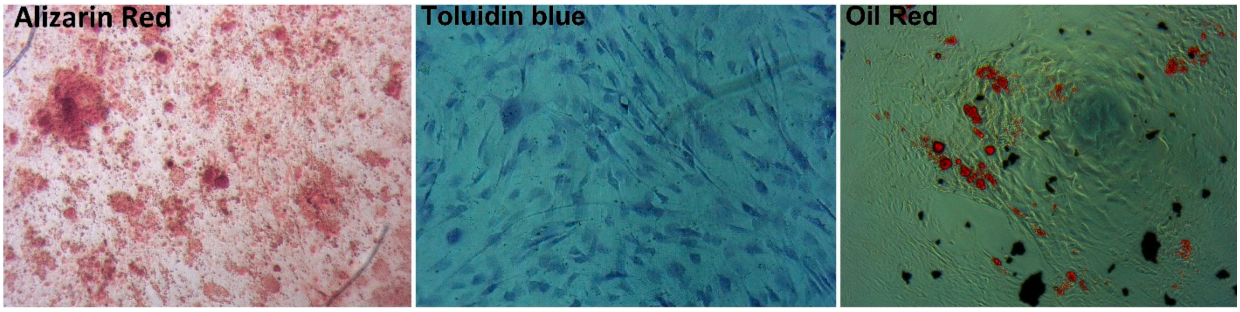

FigureS1. Micrographs of mesenchymal stromal cells (MSCs) isolated from human adipose tissue after induction of osteogenic (A), chondrogenic (B) and adipogenic (C) differentiation. Confirmation of the MSCs phenotype. Alizarin red staining of calcium deposits (A), staining of glycosaminoglycans with toluidine blue (B), and staining of lipids with Oil red O (C). Magnification x100.
